# Nordic AI-Health: a unique opportunity for discovery and clinical impact

**DOI:** 10.1016/j.lanepe.2026.101769

**Published:** 2026-07-15

**Authors:** Ole A. Andreassen, Hakon Heimer, Søren Brunak, Olli Kallioniemi, Nordic AI-Health Initiative

**Affiliations:** a Center for Precision Psychiatry, Division of Mental Health and Addiction, Oslo University Hospital, Institute of Clinical Medicine, University of Oslo, Ullevål Hospital, Building 22, P.O.Box 4956 Nydalen, 0424, Oslo, Norway; b Department of Public Health, Section for Health Data Science and AI, University of Copenhagen, Blegdamsvej 3B, Copenhagen, N 2200, Denmark; c Institute for Molecular Medicine Finland (FIMM), Helsinki Institute of Life Science (HiLIFE), University of Helsinki, P.O. Box 3 (Fabianinkatu 33), 00014, Helsinki, Finland; d Department of Oncology-Pathology, Karolinska Institutet, Stockholm, Sweden

Artificial intelligence (AI) is reshaping human activity in profound ways, especially in data- and technology-intensive fields, yet the AI impact on clinical medicine remains modest.^1^ Approved medical AI products mostly speed up clincian tasks. Translating population-scale health data into mechanistic insight, novel treatments, or genuinely personalised care has so far proved elusive.^2^ A fundamental constraint is the data itself: fragmented, siloed, and insufficiently longitudinal to train and validate models capable of capturing the full complexity of human health across a lifetime.^2^

The Nordic countries—Denmark, Finland, Iceland, Norway, Sweden, and Estonia—offer perhaps the world's most compelling setting for breaking this impasse. With a combined population exceeding 30 million served by universal healthcare, the region has spent decades compiling registry data that can be linked at the individual level. Vital events, clinical encounters, dispensed medications, social circumstances, and long-term outcomes form a longitudinal resource of unmatched depth and quality.^3^ Layered onto these registries are population-scale data from biobanks: FinnGen has enrolled roughly half a million Finns, while Amgen/deCODE has genotyped most of Iceland's adult population, adding genomic and molecular depth to the longitudinal record.^3^ High public trust and digital literacy have enabled sustained, broad-consent participation, yielding data assets that directly address the most cited barriers to AI in healthcare: fragmentation of data, privacy risk, small sample sizes, and limited validation opportunities. This Comment is a call for Nordic stakeholders to exploit these resources, as called for by the Nordic AI-Health Initiative (Andreassen et al., 2026 SSRN preprint https://papers.ssrn.com/sol3/papers.cfm?abstract_id=6688979).

Individually, each Nordic country possesses remarkable data; collectively, the region can offer sample sizes and cross-system diversity sufficient to train robust, generalisable AI foundation models and to validate findings across independent populations.^4^ Cross-Nordic aggregation is valuable for both common and rare diseases, understudied phenotypes, and equity-critical subgroup analyses. It also enables comparison of health interventions across similar but distinct healthcare environments, a form of natural experiment unavailable in more homogeneous settings.

The methodological infrastructure is within reach. Federated learning, where the model travels to each data custodian and only updated parameters are shared, allows training across distributed repositories without pooling sensitive records,^5^ aligning with GDPR and the emerging European Health Data Space (EHDS).^6^ Early validation work supports the feasibility: Delphi-2M model, a generative transformer developed on UK Biobank data, transferred successfully to Danish national registries and predicted outcomes across a wide span of disease categories.^7^ Each Nordic country already hosts substantial compute capacity, for example LUMI in Finland (co-owned with Norway), Gefion in Denmark, NORTRE in Norway, and Berzelius and MIMER in Sweden. Linking these systems under a coordinated, biomedicine-oriented framework would unlock the throughput required to train multimodal foundation models at scale.^2^

Governance and trust are critical for successful development and deployment of AI technology in healthcare.^8^ The democratic accountability and universal healthcare ethos in Nordic societies provide a natural scaffold for responsible AI. Any clinical AI output should be positioned as decision support that complements rather than substitutes for clinician judgement, with named human owners for each consequential decision and ongoing surveillance for model degradation and disparate performance across patient subgroups.^9^ Public-private partnerships, modelled on UK Biobank's transparent data access policy, would allow academia and industry, including Nordic startups, to contribute and benefit under fair, equitable terms.

There is a window of opportunity for a Nordic AI-Health initiative, and the Nordic health ministries should act now while we have the momentum. The EU AI Act and EHDS create a regulatory architecture for cross-border health data use and the Nordic region's abundant renewable energy capacity makes it well placed to host energy-intensive AI training with a smaller carbon footprint than most alternatives. The institutional scaffolding also exists: the Nordic Council of Ministers, NordForsk, and the Nordic University Hospital Alliance (NUHA) could anchor a coordinated Nordic AI-Health programme built on the pillars of: a common data model, a cross-border federated learning layer, ring-fenced national funding (a recent Finnish proposal set a benchmark of 0.2% of healthcare expenditure^10^), and demonstrator projects in disease areas where the Nordic registry–and–biobank combination delivers the greatest analytical leverage.

The Nordic region has scientific leadership in healthcare through a series of landmark population studies, groundbreaking biobank findings, and large-scale international collaborations.^3^ Extending that legacy into the AI era requires the same ingredients that made earlier successes possible: coordinated vision, sustained investment, and collaborative governance grounded in shared values (Box 1). A *Nordic AI-Health platform* would improve care for 30 million citizens, and also offer the world a proof-of-concept model for responsible, equitable AI in healthcare.

Box 1 **A unique Nordic opportunity to unlock AI-Health benefits.**

**Existing advantages for AI-Health in the Nordics**

• **Unmatched longitudinal data**—Decades of population-wide, individually linkable registry records.
• **Population-scale biobanks**—Add deep genomic and molecular data.
• **High public trust**—Broad consent and digital literacy overcome key AI data barriers.
• **Collective Nordic scale**—Pooled data enable robust, generalisable AI models across populations.
• **Favorable policy environment**—EHDS, EU AI Act, and renewable energy enable responsible AI.

**Keys to building a Nordic AI-Health ecosystem**

• **Launch Nordic AI-Health initiative** governed by health ministries, hospitals, and universities.
• **Commit dedicated national funding** benchmarked at 0.2% of each nation's healthcare expenditure.
• **Align with EHDS**, harmonizing GDPR and EU AI Act frameworks across all countries.
• **Ground initiative in transparent public-private partnerships**, modeled on UK Biobank.
• **Demonstrate impact** through coordinated pilot projects targeting high-burden diseases.

## Contributors

OAA, HH, OK, SB organized the workshop, “Nordic AI in Research and Healthcare”, at the University of Copenhagen, Denmark, in June 2025, which generated the ideas for this article. OAA and HH wrote the first draft, and SB and OH revised subsequent drafts. All authors reviewed and approved the final version of the manuscript. Generative AI tools were used to improve readability, grammar, and language.

## Declaration of interests

OAA is a consultant to Precision-Health.ai and Ledidi, and has received speaker's honorarium from Eli Lilly, Bristol Myers Squibb, H. Lundbeck, Otsuka, Sunovion, and Janssen. HH is executive officer of the Nordic Society of Human Genetics and Precision Medicine. SB reports stock ownership in Hoba Therapeutics, Novo Nordisk, H. Lundbeck, and Eli Lilly. OK is founder and shareholder in Sartar Therapeutics and received honoraria from KAW, Novo Nordisk Foundation, and Sitra.
